# Effect of cognitive task on postural control of the patients with chronic ankle sprain

**DOI:** 10.1186/1757-1146-5-S1-P24

**Published:** 2012-04-10

**Authors:** Zeinab Shiravi, Saeed Talebian, Mohammad Reza Hadian, Gholam Reza Oliaie

**Affiliations:** 1 Dept. Physiotherapy, Rehabilitation Faculty, Tehran University of Medical Sciences, Tehran, Iran

## Background

Chronic ankle instability can affect activities of daily living in patients. While many studies have indicated postural control deficits in these patients [1,2], the effect of a dual task on postural control has not been examined yet [3].

## Materials and methods

Postural stability in CAI patients (n=8) and healthy subjects (n=10) was measured using the Force Plate. Eight positions concluded two different stances (double & single) with closed or opened eyes. All positions concurrently were done with a cognitive task. Anterior/posterior (Rfa) and medial/lateral (Rsw) mean sway, area and mean velocity quantified static postural control [4].

## Results

Mean sway significantly increased in patients in the anterior/posterior (single and double leg stance) and medial/lateral (single leg stance) directions (P<0.05). While performing a dual task anterior/posterior mean sway decreases within the patients group on the impaired leg stance (P<0.05). Area significantly increased in patients in single leg stance but decreased in the bilateral standing positions except open eyes with cognitive task. Mean velocity significantly decreased (single leg stance) and increased (double leg stance). No difference is seen in the healthy subjects.

## Conclusion

Postural control deficits were identified in participants with chronic ankle instability. In view of the fact that a cognitive task resulted in decreasing displacement of centre of pressure in patients, this method may identify as an examination and a plan of treatment for affecting on ankle stabilizing factors.

## References

[B1] De Noronha M Refshauge KM Herbert RD Kilbreath SL Hertel J Do voluntary strength, proprioception, range of motion, or postural sway predict occurrence of lateral ankle sprain J Sport Med 2006 40 824 8 10.1136/bjsm.2006.029645 PMC2465053 16920769

[B2] McKoen PO Hertel J Systematic Review of Postural Control and Lateral Ankle Instability, Part1: Can deficits be detected with instrumented testing J Athl Train 2008 43 293 304 10.4085/1062-6050-43.3.293 18523566 PMC2386423

[B3] Woollocott M Shumway-cook A Attention and the control of posture and gait: a review of an emerging area of research Gait posture 2002 16 1 14 12127181 10.1016/s0966-6362(01)00156-4

[B4] Raymarkers JA Samson MM Verhaar HJ The Assessment of Body Sway and the Choice of the Stability Parameter(s) Gait Posture 2005 21 48 58 10.1016/j.gaitpost.2003.11.006 15536033

